# Joint Estimation Method of DOD and DOA of Bistatic Coprime Array MIMO Radar for Coherent Targets Based on Low-Rank Matrix Reconstruction

**DOI:** 10.3390/s22124625

**Published:** 2022-06-19

**Authors:** Zhiyuan You, Guoping Hu, Hao Zhou, Guimei Zheng

**Affiliations:** Air Defense and Antimissile School, Air Force Engineering University, Xi’an 710038, China; yzy343625419@163.com (Z.Y.); 17792611529@126.com (H.Z.); zheng-gm@163.com (G.Z.)

**Keywords:** bistatic radar, MIMO radar, coprime array, coherent signal, convex optimization

## Abstract

Based on low-rank matrix reconstruction theory, this paper proposes a joint DOD and DOA estimation method for coherent targets with bistatic coprime array MIMO radar. Unlike the conventional vectorization, the proposed method processed the coprime array with virtual sensor interpolation, which obtained a uniform linear array to generate the covariance matrix. Then, we reconstructed the Toeplitz matrix and established a matrix optimization recovery model according to the kernel norm minimization theory. Finally, the reduced dimension multiple signal classification algorithm was applied to estimate the angle of the coherent targets, with which the automatic pairing of DOD and DOA could be realized. With the same number of physical sensors, the proposed method expanded the array aperture effectively, so that the degree of freedom and angular resolution could be improved significantly for coherent signals. However, the effectiveness of the method was largely limited by the signal-to-noise ratio. The superiority and effectiveness of the method were proved using simulation experiments.

## 1. Introduction

At the beginning of the 21st century, with the development of multiple-input multiple-output (MIMO) communication theory, scholars at the Lincoln Laboratory proposed the concept of MIMO radar [1] in 2003. MIMO radar has been studied by many scholars because of its advantages in waveform diversity [2,3,4], better resolution, and extended array aperture [5,6,7]. In recent years, direction of arrival (DOA) estimation [8,9,10,11,12,13,14,15] is increasingly linked to MIMO radar. When using conventional subspace class algorithms to estimate the DOA of coherent targets, the vector of the signal subspace penetrates into the noise subspace, resulting in a deficient covariance matrix rank that is unable to accurately estimate the number of coherent signal sources. To solve the problem, experts proposed two methods; one is the non-dimension-reduction algorithm [16,17], including the frequency domain smoothing algorithm, and the other is the dimension-reduction algorithm [18], including the classical spatial smoothing algorithm and matrix reconstruction algorithm. When scholars estimated the DOA, a problem regarding unknown number of target sources was often encountered; thus, they devised an LS-MDL method for source enumeration [19]. Because a uniform linear array has small apertures, scholars proposed sparse arrays in order to improve the degrees of freedom [20]. The coprime array [21,22] has been widely studied by scholars due to its larger element spacing. Thus, the coprime array has a low mutual coupling effect and a high accuracy in target angle estimation.

However, scholars found it difficult to estimate the DOA of coherent targets by using sparse arrays because the common method of processing sparse arrays is to form a sum-difference coarray and vectorize it to obtain a virtual array, but the above method is invalid for coherent signals. Some scholars proposed a low-rank matrix-reconstruction method [23,24] that finds the appropriate constraint conditions through theoretical analysis and calculation to obtain a more accurate matrix reconstruction. Vaidyanathan, P. combined the low-rank matrix reconstruction algorithm with an underdetermined estimation of difference coarray [25], which used the low-rank matrix-reconstruction method to recover the missing observation matrix elements, and finally used the recovered matrix for DOA estimation. Liu, C. used the kernel norm minimization method to recover the value of “holes” in the difference coarray [26], which could expand the degrees of freedom of the array and improve the performance of the DOA estimation. Chen, T. applied the low-rank matrix reconstruction method to DOA estimation of a sparse array, and caused the covariance matrix that was obtained by the sparse array to recover to the normal matrix [27]. Zhou, C. proposed a matrix reconstruction algorithm based on a coprime array and used the idea of virtual sensor interpolation to construct virtual uniform arrays [28]. Zheng, Z. proposed an algorithm for DOA estimation of coherent signals that was based on coprime array interpolation and low-rank matrix recovery [29]. According to the method of coprime array interpolation and low-rank matrix recovery [28,29], we proposed a joint direction of departure (DOD) and DOA estimation method of bistatic coprime array MIMO radar for coherent targets based on low-rank matrix reconstruction. The method uses a virtual sensor interpolation algorithm and the kernel norm minimization theory to establish a matrix optimization recovery model. Under the condition of the same number of physical sensors, the method expands the array aperture and improves the array degree of freedom, angle estimation accuracy, and angular resolution. In addition, it is suitable for estimating coherent signals.

The framework of this paper is designed as follows. In Section 2, the paper deduces the math model of the bistatic coprime array MIMO radar. We elaborate on the method of interpolating virtual sensors into the “holes” of coprime array and the low-rank matrix reconstruction algorithm in Section 3. The effectiveness of the algorithm is proved by simulation experiments in Section 4. Finally, the conclusions are drawn in Section 5.

Notations: We use italicized boldface characters to represent vectors and matrices in this paper. Superscripts ${\left(.\right)}^{T}$ and ${\left(.\right)}^{H}$ represent transpose and conjugate-transpose respectively; diag[.] denotes a diagonal matrix; and ⊗ and ∘ denote the Kronecker product and the Hadamard product, respectively.

## 2. Signal Model of Coprime Array MIMO Radar

**Figure 1:**
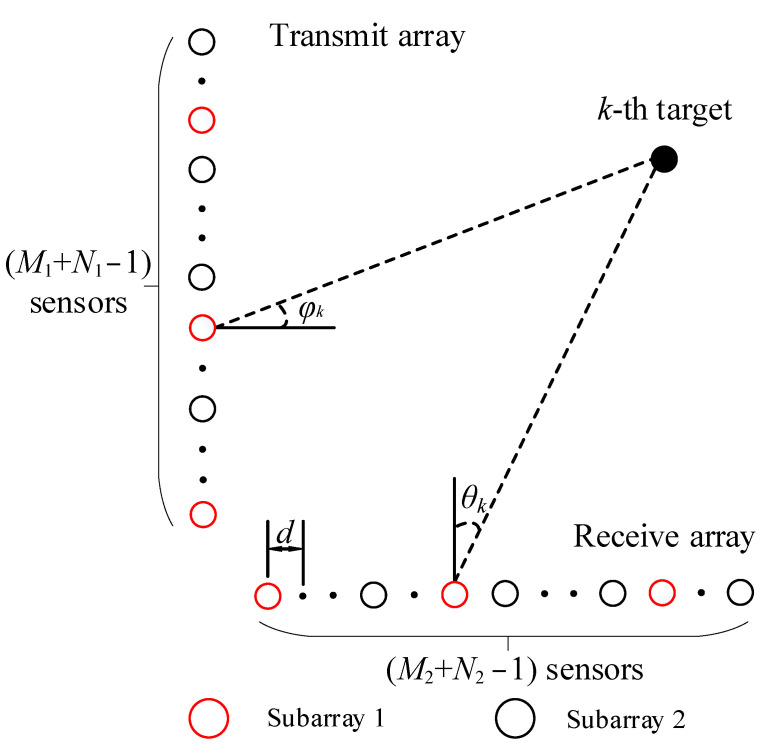
Bistatic coprime array MIMO radar model.

A coprime array is a classical sparse array structure, and it consists of two uniform linear subarrays. One subarray contains *M* sensors with an array interval *Nd*, while another sub-array contains *N* sensors with an array interval *Md*; *M* and *N* are mutually prime numbers. The bistatic coprime array MIMO radar was composed of a transmitting array and a receiving array. As shown in Figure 1, the transmitting array had $M=M_{1}+N_{1}-1$ sensors to estimate the DOD, and the receiving array had $N=M_{2}+N_{2}-1$ sensors to estimate the DOA. The black circles and red circles respectively represent the two subarrays of the coprime array; *d* represents the unit spacing, and it also represents ; λ/2; $M_{1}$ and $N_{1}$ are mutually prime numbers; and $M_{2}$ and $N_{2}$ are mutually prime numbers, ($N_{1}>M_{1}$, $N_{2}>M_{2}$). The sensors’ distribution on the transmitting array and receiving array can be given by:

(1) $$S_{t}=\{mN_{1}d|0\leq m\leq M_{1}-1\}\cup \{nM_{1}d|0\leq n\leq N_{1}-1\}$$

(2) $$S_{r}=\{mN_{2}d|0\leq m\leq M_{2}-1\}\cup \{nM_{2}d|0\leq n\leq N_{2}-1\}$$

Assuming that there are *K* coherent far-field narrowband signals in the airspace, the DOD of the *k*th signal can be represented by , $\varphi_{k}$, the DOA of the kth signal can be represented by $\theta_{k}$, the waveform vector of the *k*th signal can be represented by $s_{k}(t)$, k=1,…,K, t=1,…,L; and *L* denotes the number of snapshots of the signals. The received signal model can be given by:

(3) $$x(t)=s(t)\sum_{k=1}^{K}\alpha_{k}[a_{t}(\varphi_{k})⊗a_{r}(\theta_{k})]+n(t)\;=A\alpha s\left(t\right)+n(t)$$

where $A=A_{t}\circ A_{r}$, $\alpha =[\alpha_{1},\alpha_{2},\ldots ,\alpha_{K}]$ represents the correlation coefficient vector and $\alpha_{K}$ is nonzero known constant, $n(t)∼CN(0,\sigma_{n}^{2}I_{MN})$ represents a white noise vector with a Gaussian distribution, and $I_{MN}$ is the identity matrix of MN×MN. The steering matrices of transmitting array and receiving array are respectively given by:

(4) $$A_{t}=[a_{t}(\varphi_{1}),a_{t}(\varphi_{2}),\ldots ,a_{t}(\varphi_{K})]$$

(5) $$A_{r}=[a_{r}(\theta_{1}),a_{r}(\theta_{2}),\ldots ,a_{r}(\theta_{K})]$$

where the steering vectors $a_{t}(\varphi_{k})$ and $a_{r}(\theta_{k})$ are denoted by:

(6) $$a_{t}(\varphi_{k})={\left[1,e^{-jM_{1}\pi \sin\varphi_{k}},\ldots ,e^{-j(M_{1}(N_{1}-1)+1)\pi \sin\varphi_{k}}\right]}^{T}$$

(7) $$a_{r}(\theta_{k})={\left[1,e^{-jM_{2}\pi \sin\theta_{k}},\ldots ,e^{-j(M_{2}(N_{2}-1)+1)\pi \sin\theta_{k}}\right]}^{T}$$

The covariance matrix of the received signal can be denoted by:

(8) $$R=E[x(t)x^{H}(t)]=R_{s}A\alpha \alpha^{H}A^{H}+\sigma_{n}{}^{2}I_{MN}$$

(9) $$R_{s}=E[s(t)s^{H}(t)]=\mathrm{diag}[\sigma_{1}{}^{2},\sigma_{2}{}^{2},\ldots ,\sigma_{K}{}^{2}]$$

where $R_{s}$ represents the covariance matrix of the targets and $\sigma_{k}{}^{2}$ represents the signal power of the *k*th target.

## 3. Joint DOD and DOA Estimation Algorithm of Coherent Signals Based on Low-Rank Matrix Reconstruction

In this section, we will discuss the joint DOD and DOA estimation method of bistatic coprime MIMO radar for coherent signals. The method uses interpolated virtual array elements to convert the coprime array signal model with holes into a uniform linear array signal model without holes. It reconstructs the Toeplitz submatrix through spatial smoothing, and uses the sensor-related information of the coprime array to recovery it into a low-rank matrix through a convex optimization algorithm. Then, the full-rank covariance matrix that is reconstructed from the recovered Toeplitz submatrix is applied to the spatial spectrum estimation. Finally, the method achieves automatic pairing of the DOD and DOA of coherent targets through a dimension-reduction multiple signal classification algorithm.

### 3.1. Reconstructed Toeplitz Matrix Algorithm Based on Virtual Sensor Interpolation

**Figure 2:**
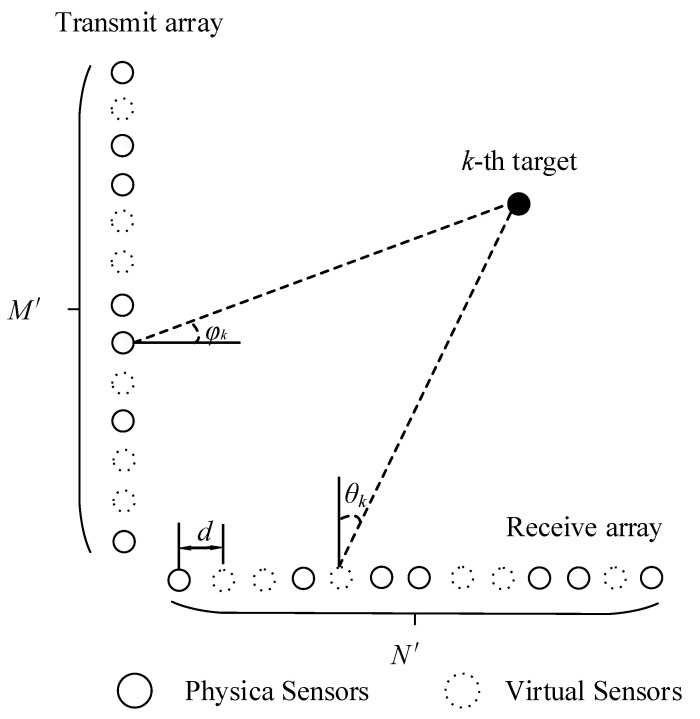
Sensor distribution of bistatic coprime array MIMO radar after interpolating virtual sensors.

As shown in Figure 1, the receiving array and transmitting array of the bistatic MIMO radar are nonuniform linear arrays, as there are holes between the physical sensors, causing the array to be discontinuous and unable to provide a covariance matrix suitable for spatial spectral estimation. The traditional method to deal with the coprime array is to obtain the sum–difference coarray of physical sensors, then vectorize the covariance matrix formed by sum–difference coarray, and finally construct the new covariance matrix to estimate the DOA. However, this method is not suitable for coherent targets. In Figure 2, the dotted circles are virtual sensors, which are later interpolated into the array, and the solid circles are physical sensors; the virtual sensors interpolated are essentially zero elements. The method fills the holes by interpolating virtual sensors [26,30,31], and a bistatic radar model of uniform linear array is constructed.

In Figure 2, the dotted circle represents the interpolation virtual sensor, which has two meanings: firstly, the virtual sensor interpolation is only implemented in the virtual domain; secondly, the virtual sensors can be used as nonfunctional sensors, and the positions of these virtual sensors are set to zeros in the array.

After interpolating the virtual sensors, the numbers of transmitting array sensors and receiving array sensors become $M^{\prime }=M_{1}(N_{1}-1)+1$ and $N^{\prime }=M_{2}(N_{2}-1)+1$, respectively. Assuming that the virtual sensors’ (dotted circles) position on the transmitting array is ${\mathbb{Z}}_{t}$ and the virtual sensors’ position on the receiving array is ${\mathbb{Z}}_{r}$, after interpolating the virtual sensors, the transmitting array sensors’ distribution and receiving array sensors’ distribution can be given by:

(10) $${S_{t}}^{\prime }=\{U_{t}\cup Z_{t}|U_{t}\in S_{t},Z_{t}\in {\mathbb{Z}}_{t}\}$$

(11) $${S_{r}}^{\prime }=\{U_{r}\cup Z_{r}|U_{r}\in S_{r},Z_{r}\in {\mathbb{Z}}_{r}\}$$

For example, assuming that the positions of the transmitting array $S_{t}$ and the receiving array $S_{r}$ sensors are both {0,3d,5d,6d,9d,10d,12d}, after interpolating the virtual sensors, the positions of the transmitting array ${S_{t}}^{\prime }$ and the receiving array ${S_{r}}^{\prime }$ sensors are both {0,‘0’,‘0’,3d,‘0’,5d,6d,‘0’,‘0’,9d,10d,‘0’,12d}, the ‘0’ only represents virtual locations and is distributed in *d*, 2*d*, 4*d*, 7*d*, 8*d*, and 11*d*.

The transmitting steering vector and the receiving steering vector after interpolating virtual sensors are given by:

(12) $${a_{t}}^{\prime }(\varphi_{k})={\left[1,\ldots ,0,\ldots ,e^{-j(M_{1}(N_{1}-1))\pi \sin\varphi_{k}}\right]}^{T}$$

(13) $${a_{r}}^{\prime }(\theta_{k})={\left[1,\ldots ,0,\ldots ,e^{-j(M_{2}(N_{2}-1))\pi \sin\theta_{k}}\right]}^{T}$$

where the zeros of the transmitting steering vector and the receiving steering vector are all distributed over virtual locations.

Using the new steering vector to form the steering matrix ${A_{t}}^{\prime }$ of the transmitting array and the steering matrix ${A_{r}}^{\prime }$ of the receiving array, the output signal model of the bistatic uniform linear array is given by:

(14) $$x^{\prime }(t)=({A_{t}}^{\prime }\circ {A_{r}}^{\prime })\alpha s(t)+n(t)$$

The covariance matrix of the bistatic uniform linear array model after interpolating the virtual sensors can be expressed as:

(15) $$R^{\prime }=E[x^{\prime }(t)x^{\prime^{H}}(t)]=R_{s}A^{\prime }\alpha \alpha^{H}A^{\prime^{H}}+\sigma_{n}{}^{2}I_{M^{\prime }N^{\prime }}$$

where $A^{\prime }$ denotes the steering matrix after interpolating virtual sensors and $I_{M^{\prime }N^{\prime }}$ denotes the $M^{\prime }N^{\prime }\times M^{\prime }N^{\prime }$ dimension identity matrix.

In practical signal processing, the covariance matrix $R^{\prime }$ usually cannot be obtained directly, and it must be approximated by *L* times of snapshot signals. The processed covariance matrix is given by:

(16) $$R_{x}=\frac{1}{L}\sum_{i=1}^{L}x^{\prime }(t)x^{\prime^{H}}(t)$$

This study mainly researched the angle estimation for coherent targets, and the covariance matrix must be a rank-deficient matrix. So, $R_{x}$ cannot be directly used for spatial spectrum estimation, and it must be processed and recovered to a full-rank matrix before it can be used normally. $R_{x}$ is a covariance matrix formed by bistatic radar, so the matrix dimension is higher, and $R_{x}$ cannot be processed using a conventional spatial smoothing algorithm. We chose a spatial smoothing algorithm based on a reconstructed Toeplitz matrix that could convert $R_{x}$ into a full-rank matrix.

$R_{x}$ is a $M^{\prime }N^{\prime }\times M^{\prime }N^{\prime }$ dimensional matrix. We chose any row of the matrix; for example, the row *r*-th, $r\in [1,M^{\prime }N^{\prime }]$, and divided the row into $N^{\prime }$ vectors with each one having a $1\times M^{\prime }$ dimension. Assuming that ${\left[R_{r}\right]}_{i}$ represents the *i*-th vector in the *r*-th row of $R_{x}$, $i\in [1,N^{\prime }]$, ${\left[r_{r}\right]}_{j}$ represents the *j*-th element in ${\left[R_{r}\right]}_{i}$,$j\in [1,M^{\prime }]$.

We performed spatial smoothing on the *r*-th row of $R_{x}$; the unit of smoothing was ${\left[R_{r}\right]}_{i}$. It could be regarded as one-dimensional spatial smoothing for $N^{\prime }$ sensors; the number of subarrays and the number of sensors in each sub-array were all $Q_{r}$, $Q_{r}=(N^{\prime }+1)/2$. The Toeplitz matrix ${R^{\prime }}_{r}$ can be constructed and expressed as:

(17) $${R^{\prime }}_{r}=\left[\begin{array}{cccc} {\left[{R^{\prime }}_{r}\right]}_{Q_{r}} & {\left[{R^{\prime }}_{r}\right]}_{Q_{r}+1} & \ldots & {\left[{R^{\prime }}_{r}\right]}_{N^{\prime }}\\ {\left[{R^{\prime }}_{r}\right]}_{Q_{r}-1} & {\left[{R^{\prime }}_{r}\right]}_{Q_{r}} & \ldots & {\left[{R^{\prime }}_{r}\right]}_{N^{\prime }-1}\\ \vdots & \vdots & \ldots & \vdots \\ {\left[{R^{\prime }}_{r}\right]}_{1} & {\left[{R^{\prime }}_{r}\right]}_{2} & \ldots & {\left[{R^{\prime }}_{r}\right]}_{Q_{r}} \end{array}\right]$$

For the Toeplitz matrix ${R^{\prime }}_{r}$, we smoothed the ${\left[{R^{\prime }}_{r}\right]}_{i}$ of in units of ${\left[r_{r}\right]}_{j}$, and it could be regarded as one-dimensional spatial smoothing for $M^{\prime }$ sensors; the number of subarrays and the number of sensors in each subarray were all $Q_{t}$, $Q_{t}=(M^{\prime }+1)/2$. The Toeplitz matrix $R_{i}$ could be constructed and is given by:

(18) $$R_{i}=\left[\begin{array}{cccc} {\left[r_{i}\right]}_{Q_{t}} & {\left[r_{i}\right]}_{Q_{t}+1} & \ldots & {\left[r_{i}\right]}_{M^{\prime }}\\ {\left[r_{i}\right]}_{Q_{t}-1} & {\left[r_{i}\right]}_{Q_{t}} & \ldots & {\left[r_{i}\right]}_{M^{\prime }-1}\\ \vdots & \vdots & \ldots & \vdots \\ {\left[r_{i}\right]}_{1} & {\left[r_{i}\right]}_{2} & \ldots & {\left[r_{i}\right]}_{Q_{t}} \end{array}\right]$$

We performed spatial smoothing twice on the *r*-th row of $R_{x}$. The first spatial smoothing obtained $Q_{r}$ Toeplitz matrices and formed ${R^{\prime }}_{r}$ in (17). For the ${\left[{R^{\prime }}_{r}\right]}_{i}$ in ${R^{\prime }}_{r}$, the second spatial smoothing obtained $Q_{t}$ Toeplitz matrices and formed $R_{i}$ in (18). Assuming that $R_{i}$ in (18) corresponded to ${\left[{R^{\prime }}_{r}\right]}_{i}$ in (17), ${R^{\prime }}_{r}$ became a new matrix $R_{x_{r}}$. Thus, the *r*-th row of $R_{x}$ can become a matrix $R_{x_{r}}$ after the above two spatial smoothings.

According to the above smoothing process, $R_{x_{r}}$ was a $Q_{r}Q_{t}\times Q_{r}Q_{t}$ dimension full-rank covariance matrix. In addition, if $M^{\prime }$ and $N^{\prime }$ were odd numbers, the matrix forms of ${R^{\prime }}_{r}$ and $R_{i}$ were the same as (17) and (18). If $M^{\prime }$ and $N^{\prime }$ were even numbers, we still used the smoothing method, but set the matrix main diagonal elements of both ${R^{\prime }}_{r}$ and $R_{i}$ to zero.

### 3.2. Joint DOD and DOA Estimation Algorithm Based on Convex Optimization to Recover Covariance Matrix

As described above, we proposed a method of interpolating virtual sensors “0” to convert the coprime array into a uniform linear array, so the covariance matrix obtained after two Toeplitz matrix reconstructions had a small zero element, and then we needed to recover the zero elements to optimal elements. This section focuses on the problem of matrix recovery, and uses the kernel norm minimization theory to establish a matrix optimization recovery model.

Assuming that the transmitting array sensors position ${S_{t}}^{\prime }$ and the receiving array sensors position ${S_{r}}^{\prime }$ can be represented as $S_{t}$ and $S_{r}$ by vectors $S_{t}\in {\mathbb{C}}^{1\times M^{\prime }}$ and $S_{r}\in {\mathbb{C}}^{1\times N^{\prime }}$, a matrix S can be represented by $S=S_{t}S_{r}{}^{T}$, and a vector ***V*** can be obtained by vectorizing S, $V\in {\mathbb{C}}^{1\times M^{\prime }N^{\prime }}$. $S_{t}$ and $S_{r}$ had some zero elements, so ***V*** similarly had zero elements, and we denoted the zero elements in ***V*** in order as F and the nonzero elements in ***V*** in order as k.

Thus, the received signal of the interpolated virtual sensors can also be given by:

(19) $$x^{\prime }(t)={\langle x^{\prime }(t)\rangle }_{i}=\{\begin{array}{l} {\langle x^{\prime }(t)\rangle }_{i},i\in k\\ 0,i\in F \end{array}$$

where *i* denotes the value in order from 1 to $M^{\prime }N^{\prime }$ and ${\langle .\rangle }_{i}$ denotes the virtual signal at *i*. If i∈F, the virtual signal is 0, and if i∈k, the virtual signal is ${\langle x^{\prime }(t)\rangle }_{i}$.

According to References [28,29], it can be seen that we needed a Toeplitz matrix to estimate the target angles, but $R_{x_{r}}$ was not a Toeplitz matrix after matrix reconstruction of (17) and (18). The matrix set $R_{i}$,$i\in [1,N^{\prime }]$ in (18) were all Toeplitz matrices, and they could be used for low-rank matrix reconstruction.

Take the 1-th matrix $R_{1}$ as an example, and build a projection matrix $P_{1}$ that matches $R_{1}$,$R_{1}\in {\mathbb{C}}^{Q_{r}\times Q_{r}}$, $P_{1}\in {\mathbb{C}}^{Q_{r}\times Q_{r}}$. The construction rules of $P_{1}$ hold that if a certain position in $R_{1}$ is 0, then the same position in $P_{1}$ is also 0, otherwise it is 1.

According to the kernel norm minimization theory in [26], we assumed that $R_{1}$ was a reference matrix, and needed to obtain an ideal matrix $R_{T_{1}}$ with low rank characteristics. $R_{T_{1}}$ represents a recovery matrix; when it was a Hermitian Toeplitz matrix, its positive semidefiniteness could be guaranteed, and then we could establish a nonconvex optimization problem with $R_{T_{1}}$ as a variable:

(20) $$\underset{R_{T_{1}}\in {\mathbb{C}}^{L\times L}}{\min}\;\mathrm{rank}(R_{T_{1}})\;s.t.\;{\left\|(R_{T_{1}}\circ P_{1})-R_{1}\right\|}_{F}^{2}<\varepsilon \;,\;R_{T_{1}}\geq 0$$

where ${\left\|.\right\|}_{F}$ represents the Frobenius norm and ε represents the error threshold that obtains $R_{T_{1}}$.

Because the rank minimization optimization problem is a nonconvex optimization problem, there may be countless local optimal solutions in the feasible region set, which is difficult to solve. In order to solve this problem, we chose convex relaxation techniques to transform the nonconvex optimization problem in (20) into a convex optimization problem that minimized the trace of ideal matrix $R_{T_{1}}$. The convex optimization problem is given by:

(21) $$\underset{R_{T_{1}}\in {\mathbb{C}}^{L\times L}}{\min}\;\mathrm{tr}(R_{T_{1}})\;s.t.\;{\left\|(R_{T_{1}}\circ P_{1})-R_{1}\right\|}_{F}^{2}<\varepsilon \;,\;R_{T_{1}}\geq 0$$

where tr(.) represents the trace of the matrix; (21) can also be represented as:

(22) $$\underset{R_{T_{1}}\in {\mathbb{C}}^{L\times L}}{\min}\frac{1}{2}{\left\|(R_{T_{1}}\circ P_{1})-R_{1}\right\|}_{F}^{2}+\mu \mathrm{tr}(R_{T_{1}})\;s.t.\;R_{T_{1}}\geq 0$$

where μ represents the error of $R_{T_{1}}$.

We could obtain an ideal Hermitian Toeplitz matrix $R_{T_{1}}$ using (22), and $R_{T_{1}}$ was the solution of the first matrix in $R_{i}$, $i\in [1,N^{\prime }]$. If all the matrices in the matrix set $R_{i}$, $i\in [1,N^{\prime }]$ were solved by convex optimization, then we could obtain an ideal matrix set $R_{T_{i}}$,$i\in [1,N^{\prime }]$. We processed all matrices in $R_{T_{i}}$ using (17) and obtained a covariance matrix $R_{x_{r}}$ to estimate the target angles.

In order to further improve the utilization of covariance matrix information and improve the accuracy and resolution of angle estimations, we chose all the rows in $R_{x}$, processed the information in the $M^{\prime }N^{\prime }$ rows, and took the average to obtain a covariance matrix $R_{xx}$ that estimated the spatial spectral information:

(23) $$R_{xx}=\sum_{r=1}^{M^{\prime }N^{\prime }}\frac{1}{M^{\prime }N^{\prime }}R_{x_{r}}$$

In terms of the spatial spectrum estimation algorithm, we chose the RD-MUSIC algorithm [32]. The RD-MUSIC algorithm not only avoids the high complexity of the 2D-MUSIC algorithm, but also has a better performance compared with the ESPRIT algorithm [33]. Moreover, the RD-MUSIC algorithm can automatically match the DOD and DOA without additional angle pairing.

The spatial spectrum estimation function is given by:

(24) $$f=\frac{1}{A_{1}{}^{H}E_{n}E_{n}^{H}A_{1}}$$

where $A_{1}={a_{t}}^{\prime }(\varphi )⊗{a_{r}}^{\prime }(\theta )$,$E_{n}$ represent the signal subspace of $R_{xx}$. Equation (24) is also expressed as:

(25) $$f=\frac{1}{{a_{r}}^{\prime }{\left(\theta \right)}^{H}V(\varphi ){a_{r}}^{\prime }(\theta )}$$

According to [29], the DOA and DOD of the *k*th target can be given by:

(26) $${\hat{\theta }}_{k}=\arg\min\frac{1}{e_{1}^{T}V{\left(\varphi_{k}\right)}^{-1}e_{1}}=\arg\max(e_{1}^{T}V{\left(\varphi_{k}\right)}^{-1}e_{1})$$

(27) $${\hat{\varphi }}_{k}=\arg\min\frac{1}{e_{1}^{T}V{\left(\theta_{k}\right)}^{-1}e_{1}}=\arg\max(e_{1}^{T}V{\left(\theta_{k}\right)}^{-1}e_{1})$$

where $e_{1}={\left[1,0,\ldots ,0\right]}^{T}$.

The method steps proposed in this section are represented in Algorithm 1.
**Algorithm 1: The steps of algorithm are as follows:**Input: received signal: *x*(t), t = 1,2, …, *L*;Output: $\{{\hat{\varphi }}_{k},{\hat{\theta }}_{k}\},k=1,2,\ldots ,K$;Step:   1: Build the covariance matrix $R_{x}$ based on interpolating virtual sensors as in (16);   2: Perform smooth reconstructions of the Toeplitz matrix for any row of $R_{x}$ to obtain matrix set $R_{i}$;   3: Recover the zero elements in $R_{i}$ through convex optimization to obtain ideal matrix set $R_{T_{i}}$ as in (22);   4: Smooth $R_{T_{i}}$ again to form a covariance matrix and perform average to obtain $R_{xx}$ as in (23);   5: Estimate $\left\{{\hat{\varphi }}_{k},{\hat{\theta }}_{k}\right\}$ with RD-MUSIC;

## 4. Simulation Results and Analysis

Assuming that the experiment was carried out under the condition of ideal white noise, and the estimated targets were all coherent signals, $M_{1}=M_{2}=3$ and $N_{1}=N_{2}=5$. The positions of the transmitting array and the receiving array sensors are both {0,3d,5d,6d,9d,10d,12d}, the number of transmitting array and receiving array subarrays sensors were all 7, and the value of μ was 0.025.

This section proved the effectiveness of the algorithm proposed in this paper by comparing the degrees of freedom performance, spatial spectrum performance, angular resolution performance, and root-mean-square error (RMSE) of different algorithms under the condition of the same physical sensors. The existing methods for estimating coherent targets’ DOD and DOA are all based the uniform linear array, so we chose a low-rank matrix reconstruction (LMR) algorithm in this study, and used the transmit/receive diversity smoothing (TRDS) algorithm in [34,35], the asymmetric spatial difference smoothing (ASDS) algorithm in [36], and a traditional spatial smoothing (SMS) algorithm for comparison.

### 4.1. Number of Estimated Targets

In this part, we assumed that the number of snapshots was 200 and the signal-to-noise ratio (SNR) was 10 dB. There were 10 fully coherent targets in the airspace, and their positions were distributed over the range [−60°, 75°], where the DODs and DOAs were all located at [−60°, −45°, −30°, −15°, 0°, 15°, 30°, 45°, 60°, 75°].

**Figure 3:**
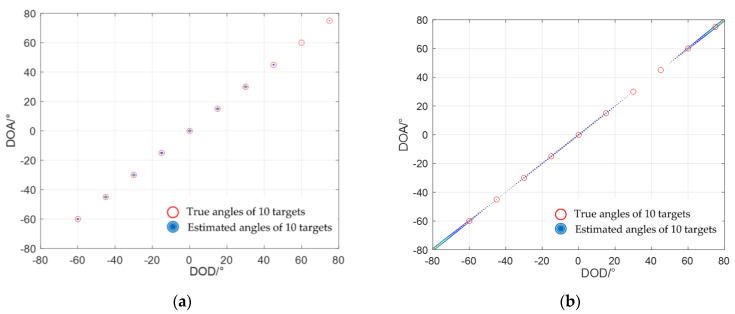

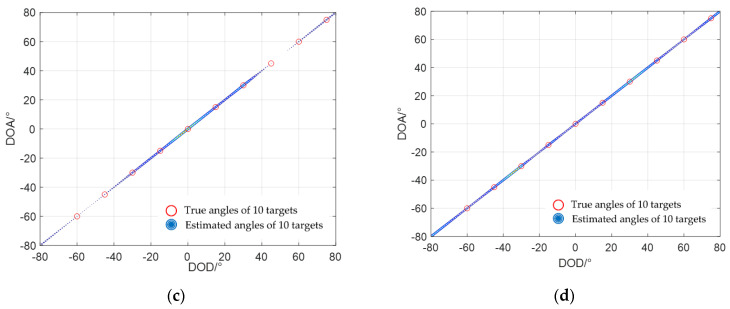
Results of estimating 10 targets by different algorithms: (**a**) LMR algorithm; (**b**) SMS algorithm; (**c**) ASDS algorithm; (**d**) TRDS algorithm.

Figure 3 shows the spatial spectrum contour of the DOD and DOA joint estimation for coherent targets. The red circles denote the true angles of 10 targets, and the spectral peak contour denotes the estimated angles of 10 targets. The four figures in Figure 3 are as follows: (a) LMR algorithm; (b) SMS algorithm; (c) ASDS algorithm; and (d) TRDS algorithm. Figure 3a shows the spectral peak contour completely overlapped by the red circles, so we could clearly estimate 10 targets using the LMR algorithm. However, the spatial peaks overlap each other in the other figures, and it was difficult to estimate all target numbers using another algorithm. So, the simulation results in this part proved the effectiveness of the LMR algorithm in the performance of the degrees of freedom.

### 4.2. Spatial Spectrum Estimation

In this part, we assumed that the number of snapshots was 200 and the SNR was 10 dB. The DODs of targets were $\left\{50^{\circ },5^{\circ },15^{\circ },25^{\circ },45^{\circ },25^{\circ }\right\}$, the DOAs of targets were $\left\{15^{\circ },20^{\circ },50^{\circ },10^{\circ },35^{\circ },35^{\circ }\right\}$, and the estimated targets were six fully coherent signals.

**Figure 4:**
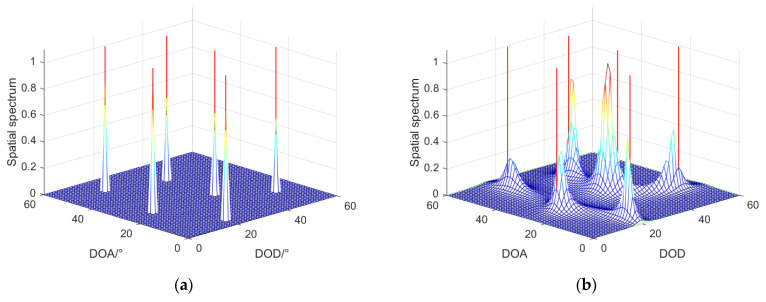

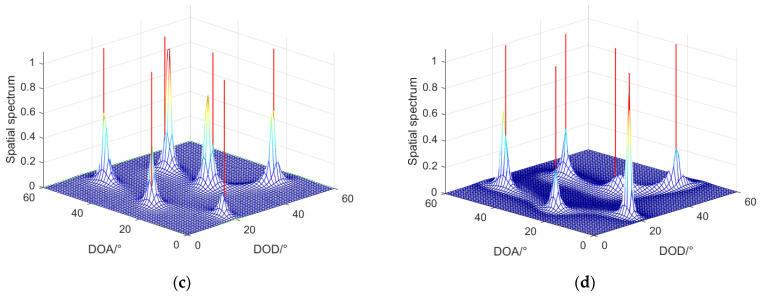
Results of the spatial spectral estimation of different algorithms: (**a**) LMR algorithm; (**b**) SMS algorithm; (**c**) ASDS algorithm; (**d**) TRDS algorithm.

Figure 4 shows the spatial spectrum of the DOD and DOA joint estimation for the coherent targets. The red lines denote the true angles of six targets, and the spectral peak denotes the estimated angles of six targets. The four figures in Figure 4 are as follows: (a) LMR algorithm; (b) SMS algorithm; (c) ASDS algorithm; and (d) TRDS algorithm. Figure 4a shows that the spatial spectral peaks completely overlapped with the red line, so we could use the LMR algorithm to obtain a clear spatial spectral peak map. However, the spatial spectral peaks of other figures had difficulty corresponding to the red line, and some spatial spectral peaks even overlap each other in Figure 4b. Thus, the simulation results in this part proved the effectiveness of the LMR algorithm in the performance of the spatial spectral estimation.

### 4.3. Angular Resolution

In this part, we assumed that the number of snapshots was 200 and the SNR was 10 dB. The DODs and DOAs of targets were $\left(\theta_{1},\varphi_{1})=(4.5^{\circ },4.5^{\circ }\right)$, $\left(\theta_{2},\varphi_{2})=(7^{\circ },7^{\circ }\right)$; and the estimated targets were two fully coherent signals.

**Figure 5:**
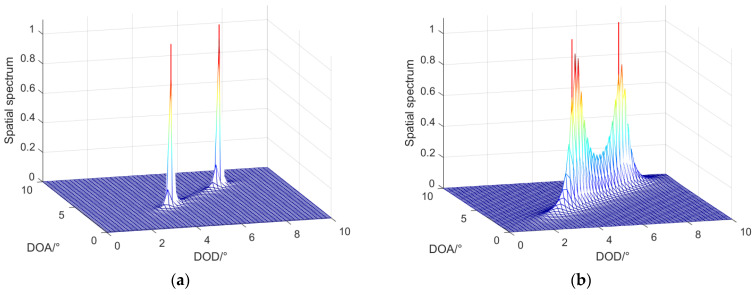

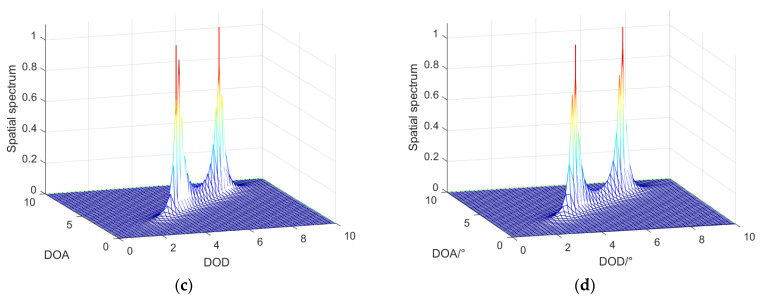
Results of the angular resolution of different algorithms: (**a**) LMR algorithm; (**b**) SMS algorithm; (**c**) ASDS algorithm; (**d**) TRDS algorithm.

Figure 5 shows the spatial spectral peaks of two adjacent targets, and also reveals the performance in angular resolution of the four algorithms. The red lines denote the true angles of two targets, and the spectral peak denotes the estimated angles of two targets. Under the condition of high SNR, we could use the LMR algorithm to estimate two adjacent targets, and there is no overlap between normalized spectral peaks in Figure 5a. We could use the TRDS algorithm and the ASDS algorithm to distinguish that there were two targets, but the resolution performance was poor. However, the spatial spectral peaks obtained using the SMS algorithm had some overlapping phenomena, so it was impossible to distinguish the existence of two targets, and the angular resolution performance was not as good as that of the other algorithms. Thus, the simulation results of this part proved the effectiveness of the LMR algorithm in the performance of angular resolution.

### 4.4. Root-Mean-Square Error (RMSE)

We compared the RMSEs of different algorithms. The RMSE is a common standard that reflects the accuracy of angle estimation, and the average RMSE is defined by:

(28) $$\mathrm{RMSE}=\sqrt{\frac{1}{2\times QK}\sum_{i=1}^{Q}\sum_{k=1}^{K}\left[{\left({\hat{\varphi }}_{k}^{i}-\varphi_{k}\right)}^{2}+{\left({\hat{\theta }}_{k}^{i}-\theta_{k}\right)}^{2}\right]}$$

where *Q* denotes the number of Monte Carlo simulation times, *K* denotes the number of targets, and $\left({\hat{\varphi }}_{k}^{i},{\hat{\theta }}_{k}^{i}\right)$ denotes the estimated DOD and DOA of the *k*th target for the *i-*th Monte Carlo simulation (*i* = 1,2, …, *Q*).

**Figure 6:**
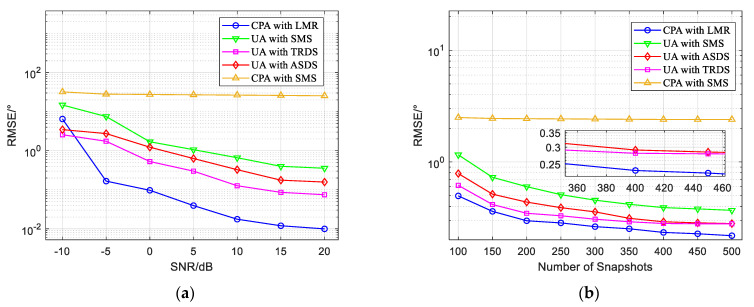
RMSE versus SNR and number of snapshots for different algorithms. (**a**) RMSE versus SNR for different algorithms; (**b**) RMSE versus number of snapshots for different algorithms.

We assumed that there were three targets in space, all of which were fully coherent signals, and their DODs and DOAs were $\left(\theta_{1},\varphi_{1})=(14^{\circ },18^{\circ }\right)$, $\left(\theta_{2},\varphi_{2})=(27^{\circ },35^{\circ }\right)$, $\left(\theta_{3},\varphi_{3})=(36^{\circ },47^{\circ }\right)$, and ; *Q* = 200. Figure 6 shows the relationship of the RMSE with the SNR and number of snapshots. The comparative algorithms used in this part were an LMR algorithm based on a coprime array, an SMS algorithm based on a coprime array, a TRDS algorithm based on a uniform linear array, an ASDS algorithm based on a uniform linear array, and an SMS algorithm based on a uniform linear array.

Figure 6a depicts the variation in the RMSE curve with the SNR, where the number of snapshots was set as 200. The LMR algorithm based on a coprime array had a higher estimation accuracy than the other algorithms under the condition of a high SNR, but its estimation performance was not satisfactory in low SNR conditions. Figure 6b depicts the variation in the RMSE curve with the number of snapshots, where the SNR was set as 10. The LMR algorithm had a high estimation accuracy with a different number of snapshots. In order to demonstrate the advantage of the proposed method for estimating coherent targets, we compared it to the SMS algorithm based on a coprime array, as shown in Figure 6. We found that using the SMS algorithm based on a coprime array resulted in a poor estimation performance, which proved it could not estimate coherent signals.

### 4.5. Time Complexity

**Figure 7:**
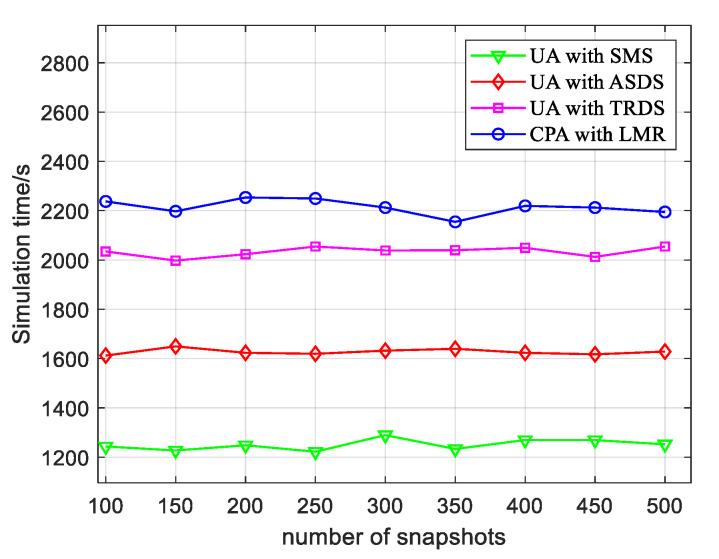
Simulation time versus number of snapshots for different algorithms.

According to the previous simulation, the LMR algorithm proposed in this paper was better than other algorithms in terms of the number of estimated targets, angular resolution, and estimation accuracy. Due to the addition of a convex optimization algorithm, the complexity of the LMR algorithm was higher than that of the other algorithms. In terms of time complexity, when 200 Monte Carlo simulation experiments were performed, Figure 7 represents a comparison of the time complexity of different algorithms with the different number of snapshots. As presented in Figure 7, the simulation showed that the LMR algorithm could achieve the effect of enhancing the performance of angle estimation by adding a small amount of computation.

## 5. Conclusions

We proposed a joint DOD and DOA estimation method of bistatic coprime array MIMO radar for coherent targets based on low-rank matrix reconstruction. The method solved the problem regarding bistatic MIMO radar based on coprime arrays being unable to estimate coherent signals, and used fewer physical sensors to achieve a better performance in the estimation. The simulation results showed that the method performed better than the existing methods in terms of the degrees of freedom performance, spatial spectrum performance, angular resolution performance, and RMSE. However, the disadvantage of this method was that it performed poorly under the condition of a low SNR due to the limitations of the convex optimization algorithm.

## Data Availability

The processed data required to reproduce these findings cannot be shared at this time, as the data also forms part of an ongoing study.
